# Crystal structures, Hirshfeld surface analysis and inter­action energies of (*Z*)-2-(4-methyl­benzylidene)- and (*Z*)-2-(furfuryl­idene)-2*H*-benzo[*b*][1,4]thia­zin-3(4*H*)-one

**DOI:** 10.1107/S2056989025008904

**Published:** 2025-10-24

**Authors:** Brahim Hni, Ahmed Moussaif, Kostiantyn V. Domasevitch, Joel T. Mague, Ahmed Mazzah, El Mokhtar Essassi, Nada Kheira Sebbar

**Affiliations:** a Laboratory of Heterocyclic Organic Chemistry Medicines Science Research Center Pharmacochemistry Competence Center Mohammed V University in Rabat Faculté des Sciences Av Ibn Battouta BP 1014 Rabat Morocco; b National Center for Nuclear Energy, Science and Technology,Rabat Morocco; c Inorganic Chemistry Department, National Taras Shevchenko National University of Kyïv, Volodymyrska Str. 64/13, Kyïv 01601, Ukraine; d Department of Chemistry, Tulane University, New Orleans, LA 70118, USA; e Science and Technology of Lille USR 3290, Villeneuve d’ascq cedex, France; University of Massachusetts Dartmouth, USA

**Keywords:** benzo-1,4-thia­zin-3-one, supra­molecular synthons, π-stacking, weak hydrogen bond, crystal structure

## Abstract

Two new 2-aryl­methyl­idene derivatives of benzo-1,4-thia­zin-3-one, namely, (*Z*)-2-(4-methyl­benzyl­idene)-2*H*-benzo[*b*][1,4]thia­zin-3(4*H*)-one, C_16_H_13_NOS, **1**, and (*Z*)-2-(furan-2-yl­methyl­idene)-2*H*-benzo[*b*][1,4]thia­zin-3(4*H*)-one, C_13_H_9_NO_2_S, **2**, are rare examples of a nearly planar structure of the 1,4-thia­zin-3-one core stabilized by conjugation. Their supra­molecular structures are very similar, being dominated by assembly of inversion dimers through highly directional reciprocal N—H⋯O bonds [N⋯O = 2.822 (2) Å for **1**; 2.881 (3) Å for **2**]. Weaker forces are represented by C—H⋯O, C—H⋯π and stacking inter­actions, with more inter­actions in the case of furfuryl­idene **2**, and they are important for consolidation of the structures. This is consistent with the results of Hirshfeld surface analysis and calculated inter­action energies. Doubling the number of O atoms, when moving from **1** to **2**, results in even larger increase in fractions of O⋯H/H⋯O contacts [7.6 to 18.6%] due to extensive inter­actions with the furyl-O acceptor and this contributes to higher packing index in the case of **2**. The far superior energetics in the structures are related with the formation of hydrogen-bonded dimers [−73.3 and −72.9 kJ mol^−1^, for **1** and **2**, respectively], followed by dispersion forces and weak C—H⋯O bonding. Identification of reliable 1,4-thia­zin-3-one based supra­molecular synthons is important for selective targeting for biomedical applications.

## Abstract 2

The crystal structures of two new (*Z*)-2-aryl­methyl­idene-2*H*-benzo[*b*][1,4]thia­zin-3(4*H*)-ones are dominated by mutual hydrogen bonding with the assembly of dimers, which may provide a prototype for reliable supra­molecular synthons with the appropriately functionalized substrates for biomedical systems.

## 1. Chemical context

Heterocyclic compounds containing both sulfur and nitro­gen atoms have garnered considerable inter­est due to their wide range of biological activities (Sebbar *et al.*, 2016*a* ▸, 2020*a* ▸; Armenise *et al.*, 2012 ▸). Among these, the 1,4-benzo­thia­zine ring system stands out as a privileged scaffold in medicinal chemistry (Trapani *et al.*, 1985 ▸; Gupta *et al.*, 1985 ▸). Its distinctive physicochemical properties allow for versatile chemical modifications and intense inter­actions with biological targets (Tawada *et al.*, 1990 ▸; Sebbar *et al.*, 2020*b* ▸). As a result, numerous 1,4-benzo­thia­zine derivatives have exhibited significant pharmacological properties, including anti-oxidant (Zia-ur-Rehman *et al.*, 2009 ▸), anti­pyretic (Warren *et al.*, 1987 ▸), anti-cancer (Gupta *et al.*, 1991 ▸) and anti-inflammatory activities (Gowda *et al.*, 2011 ▸). 1,4-Benzo­thia­zine compounds have also demonstrated their effectiveness as inter­mediates in the synthesis of new bioactive and anti-corrosion derivatives (Sebbar *et al.*, 2015 ▸; Ellouz *et al.*, 2017*a* ▸; Hni *et al.*, 2019*a* ▸) possessing anti-diabetic (Tawada *et al.*, 1990 ▸) and anti-corrosion activities (Sebbar *et al.*, 2016*b* ▸; Hni *et al.*, 2019*b* ▸; Ellouz *et al.*, 2017*b* ▸, 2018 ▸). Given the promising applications of 1,4-benzo­thia­zine derivatives, we undertook the synthesis of new compounds belonging to this class. To this end 3,4-di­hydro-2*H*-1,4-benzo­thia­zin-3-one were condensed with 4-methyl­benzaldehyde, **2a**, or furan-2-carbaldehyde, respectively, in the presence of excess sodium methoxide in di­methyl­formamide (DMF). The resulting products (*Z*)-2-(4-methyl­benzyl­idene)-2-*H*-benzo[*b*][1,4]thia­zin-3(4*H*)-one, **1**, and (*Z*)-2-(furfuryl­idene)-3,4-di­hydro-2*H*-1,4-benzo­thia­zin-3-one, **2**, were characterized by single-crystal X-ray diffraction and Hirshfeld surface analysis.
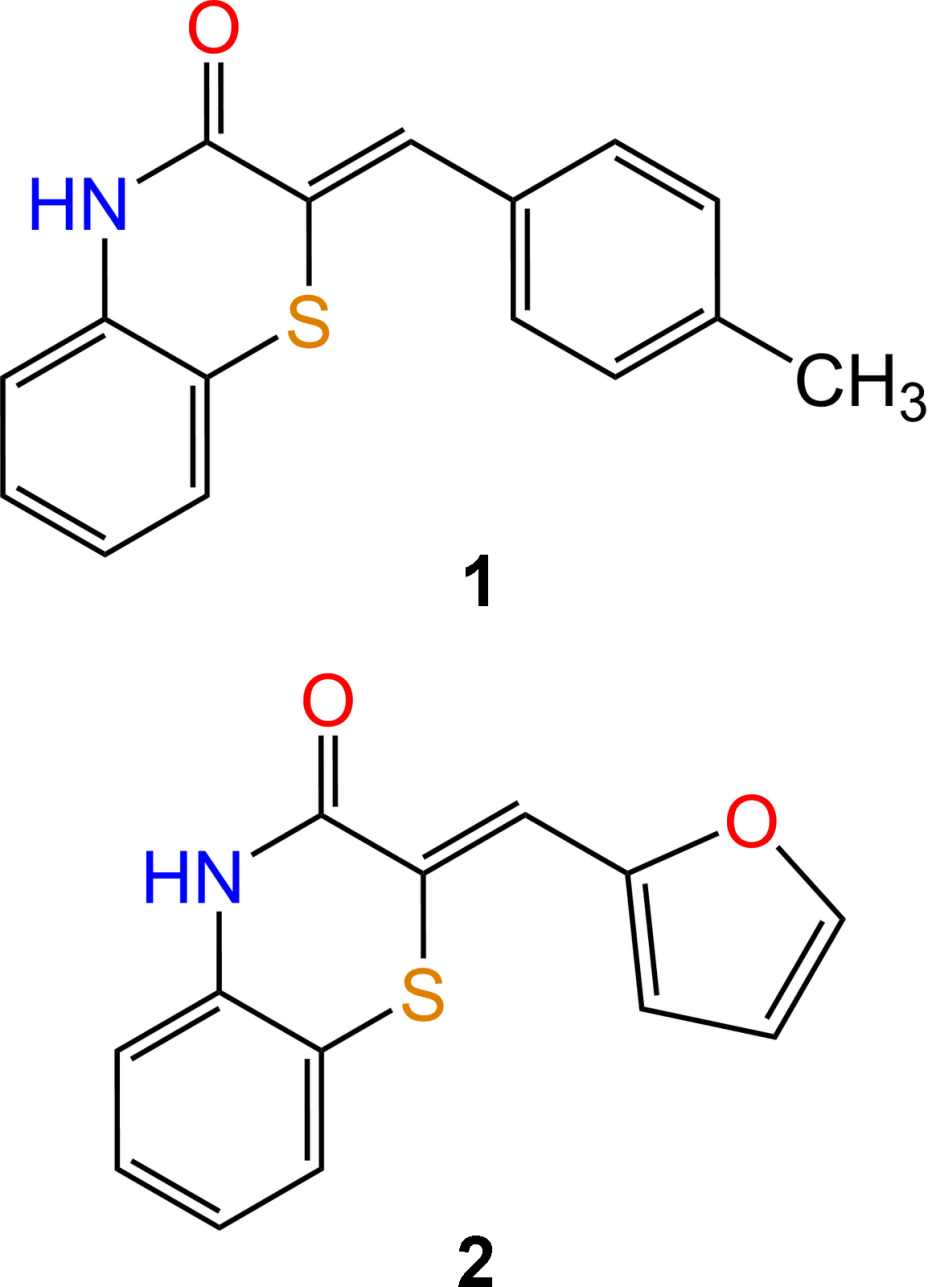


## 2. Structural commentary

**Figure 1:**
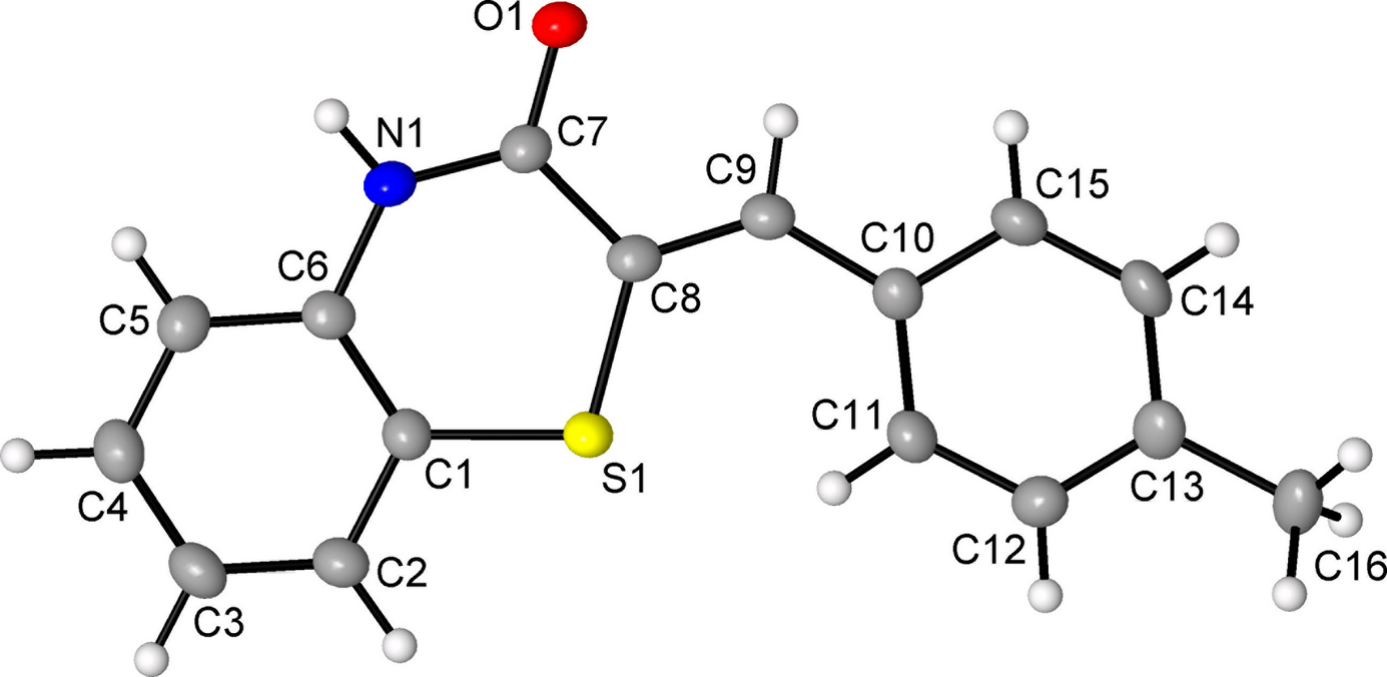
The mol­ecular structure of compound **1**, with atom labelling and displacement ellipsoids drawn at the 50% probability level.

The mol­ecular structures of the title compounds **1** and **2** are shown in Figs. 1 ▸ and 2 ▸, respectively. Both of them adopt a Z-configuration about the ethene bond. In **1**, the planes of the two aromatic counterparts, namely 1,4-benzo­thia­zin-3(4*H*)-one and tolyl, sustain a relatively small dihedral angle of 11.09 (10)°, whereas in **2** the coplanar configuration of two ring systems is violated more significantly and an appreciable inter­planar angle of 26.85 (9)° is developed due to the twist about C9—C10 bond with torsion angle C8—C9—C10—C11 being 18.1 (4)°. The most salient feature of the mol­ecular frameworks is the nearly planar geometries of the benzo­thia­zinone cores themselves. The r.m.s. deviations from the S1/N1/C1/C6–C8 planes are 0.0247 (13) Å for **1** and 0.0482 (15) Å for **2**, and such flattening has only few precedents, for example 2-(nitro­methyl­idene)-2*H*-1,4-benzo­thia­zin-3(4*H*)-one (Berestovskaya *et al.*, 2006 ▸). Any analogs with *sp*^3^ ring atoms C^2^ adopt a half-chair conformation due to loss of conjugation along S^1^—C^2^—C^3^(O)—N^4^ chain and they exist rather as cyclic amides, *e.g.* the 2*H*-1,4-benzo­thia­zin-3(4*H*)-one with a dihedral angle between the S^1^/C^9^/C^10^ and N^4^/C^3^/O/C^2^ planes φ = 22.7° (Mague & Ouzidan, 2024 ▸). The latter parameter is more informative than the usually considered bent angle along the S^1^⋯N^4^ axis, being indicative of the amide group involvement in the conjugation. For the title compounds **1** and **2**, the angles φ subtended by the S1/C1/C6 and N1/C7/O1/C8 planes are 3.36 (12) and 5.75 (14)°, respectively, and they suggest nearly coplanar arrangements. More inter­esting that N-substitution of 2-(methyl­idene)-species has the same destructive impact on conjugation as the involvement of the ring C*sp*^3^ atom. In this way, the heteroring in the 4-hexyl analog of **1** is non-planar to the same extent as in the above non-aromatic 2*H*-1,4-benzo­thia­zin-3(4*H*)-one [φ = 20.85°; Sebbar *et al.*, 2020*b* ▸]. One can postulate that the essential penalty to the conjugation in these 4-*R*-substituted species originates in steric *peri*-inter­action with the 4-*R* group, similarly to dearomatization of 1-methyl-2-quinolones by *peri*-substituents (Chen *et al.*, 2013 ▸). In the present case, the ring may be more sensitive to such factor since even 5-*H* species experience this effect and therefore the category of planar 1,4-benzo­thia­zin-3(4*H*)-ones is restricted to 2-(methyl­idene)- and N-unsubstituted species. The appreciable enhancement of conjugation in the title compounds when compared to their 4-substituted analogs is also visible from bond lengths in the C6—N1—C7—O1 chain. In particular, the C6—N1 and N1—C7 bonds [1.401 (2), 1.350 (3) and 1.409 (3), 1.342 (3) Å for **1** and **2**, respectively] are both shorter than in the 4-hexyl analog of **1** [1.4207 (17) and 1.3687 (17) Å; Sebbar *et al.*, 2020*a* ▸], and this coincides with a certain elongation of the C7=O1 bonds, which are 1.2310 (15) in the latter case, but 1.242 (2) and 1.242 (3) Å in **1** and **2**, respectively.

## 3. Supra­molecular features

**Figure 3:**
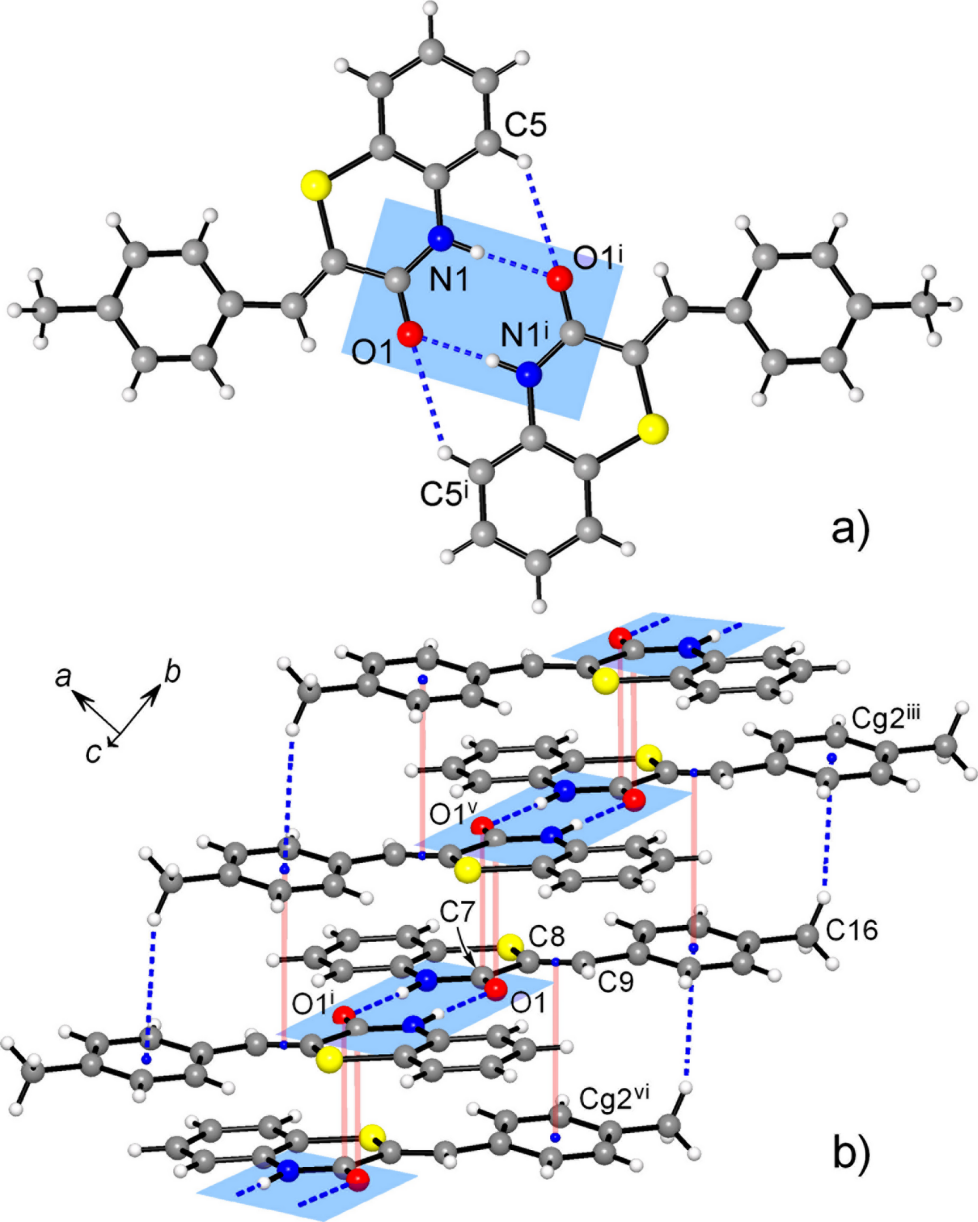
(*a*) Inversion-related hydrogen-bonded dimers in the structure of **1**; (*b*) Slipped stacking of the dimers generates columns along the *b*-axis direction, with a set of π–π and C—H⋯π inter­actions indicated in red and blue, respectively. [Symmetry codes: (i) −*x* + 1, −*y*, −*z* + 1; (iii) *x*, *y* + 1, *z*; (v) −*x* + 1, −*y* + 1, −*z* + 1; (vi) *x*, *y* − 1, *z.*]

The closely related supra­molecular structures of the title compounds are primarily governed by relatively strong hydrogen bonding accompanied with a set of weak hydrogen bonds and stacking inter­actions. Two mutual N1—H⋯O1^i^ bonds complemented by a pair of secondary C5—H⋯O1^i^ bonds assemble the mol­ecules into the inversion dimers [symmetry code (i) for **1**: −*x* + 1, −*y*, −*z* + 1; for **2**: −*x* + 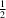, −*y* + 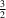, −*z* + 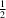] (Fig. 3 ▸). The formation of such dimers dominates the crystal chemistry of many amide-related species, with a median of N⋯O length distribution at 2.95 Å (McMahon *et al.*, 2005 ▸). In the present cases these distances are shorter [2.822 (2) and 2.881 (3) Å for **1** and **2**, respectively; Tables 1 ▸ and 2 ▸], as a consequence of stronger inter­actions between more polarized donors and acceptors NH^δ+^ C O^δ-^, similarly to an even stronger bonding of 2-pyridone in its monoclinic polymorph [2.745 (2) and 2.792 (2) Å; Arman *et al.*, 2009 ▸]. With respect to the combined N—H⋯O and C—H⋯O bonding, the observed dimers may be best related to a similar motif in α-thia­zine-indigo [N⋯O = 2.828 (3) and C⋯O = 3.492 (5) Å; Buchsbaum *et al.*, 2011 ▸]. At the same time, the comparable mol­ecular configurations of 2*H*-benzo[*b*][1,4]thia­zin-3(4*H*)-one 1,1-dioxide (Irrou *et al.*, 2023 ▸) and thio­morpholin-3-one (Ramasubbu *et al.*, 1988 ▸) do not support formation of dimers. These hydrogen-bonding preferences of benzo­thia­zinones are inter­esting in view of their selective targeting of Ser293 in the active region of acetyl­choline esterase (Haji Ali *et al.*, 2022 ▸).

**Figure 4:**
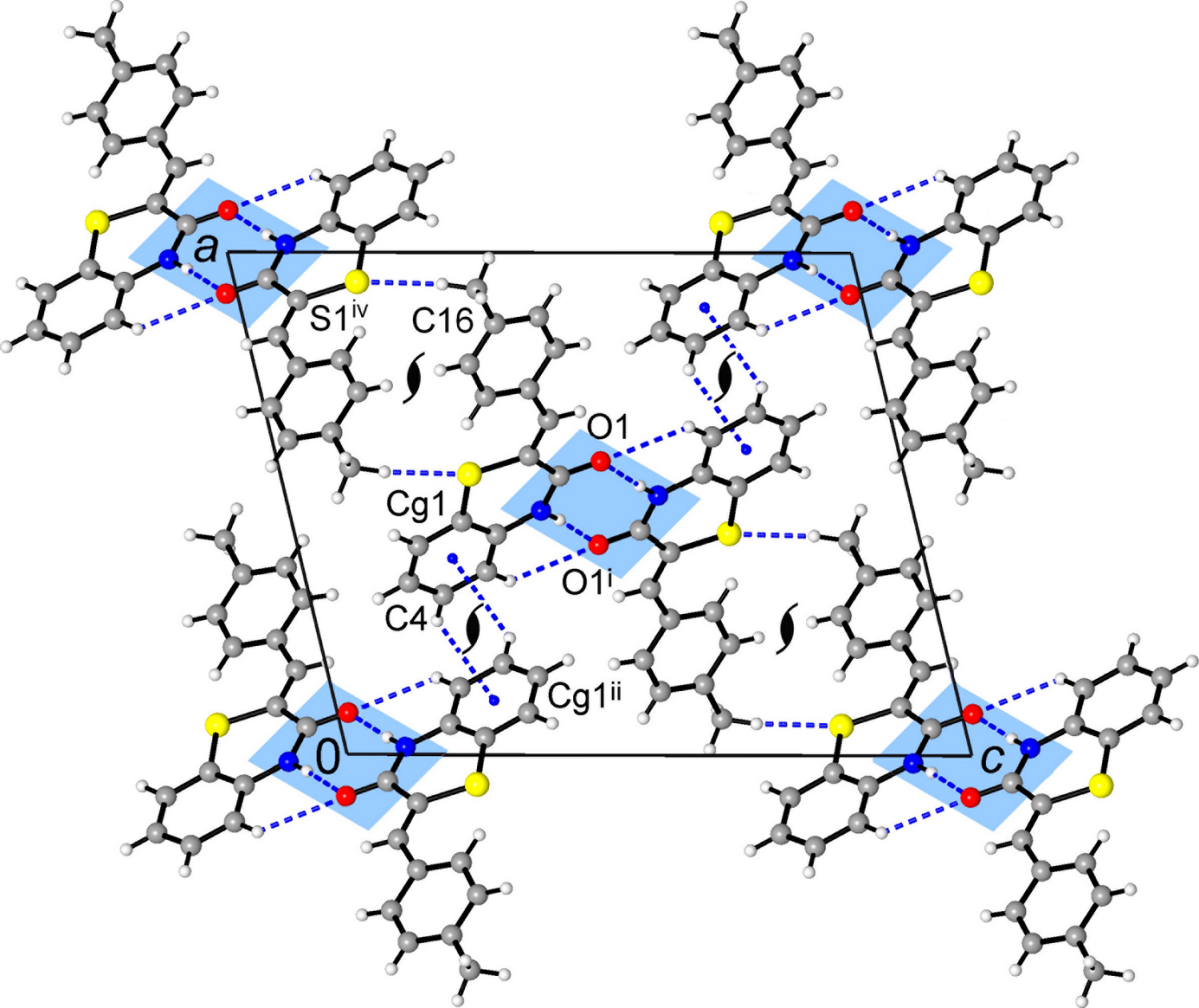
Projection of the structure **1** on the *ac* plane, showing mutual C—H⋯π bonding between columns of stacked dimers (which are orthogonal to the drawing plane) and weak inter­layer C—H⋯S inter­actions. Both kinds of bonds generate helical motifs identified here by orthogonal 2_1_ axes. [Symmetry codes: (i) −*x* + 1, −*y*, −*z* + 1; (ii) −*x* + 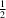, *y* − 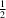, −*z* + 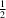; (iv) −*x* + 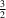, *y* + 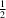, −*z* + 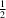.]

In **1**, the dimers are further stacked into the columns along the *b*-axis direction (Fig. 3 ▸). Within the column, one can distinguish mutual inter­actions of carbonyl groups, which are similar to lone-pair–π hole bonding in nitro compounds [C7⋯O1^iv^ = 3.173 (3) Å; symmetry code: (iv) −*x* + 1, −*y* + 1, −*z* + 1] and π–π inter­actions of ethyl­ene fragments and outer tolyl rings with a shortest contact C8⋯C11^vi^ = 3.447 (3) Å and a *Cg*3⋯*Cg*2^vi^ distance of 3.680 (2) Å [*Cg*2 and *Cg*3 are the centroids of the C10–C15 and C8/C9 groups, respectively; symmetry code (vi) *x*, *y* − 1, *z*]. In addition, two tolyl groups of translationally related mol­ecules afford very weak, but directional C—H⋯π bonds [C16⋯*Cg*2^iii^ = 3.813 (3) Å, C—H⋯*Cg*2^iii^ = 158 (2)°; symmetry code: (iii) *x*, *y* + 1, *z*]. Similar in nature C—H⋯π inter­actions between carbo rings of the benzo­thia­zinone moieties [C4⋯*Cg*(C1–C6) = 3.687 (3) Å; Table 1 ▸] generate 2_1_-helices along the *b*-axis direction, which connect the above columns into the layers parallel to the (10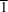) plane (Fig. 4 ▸).

**Figure 5:**
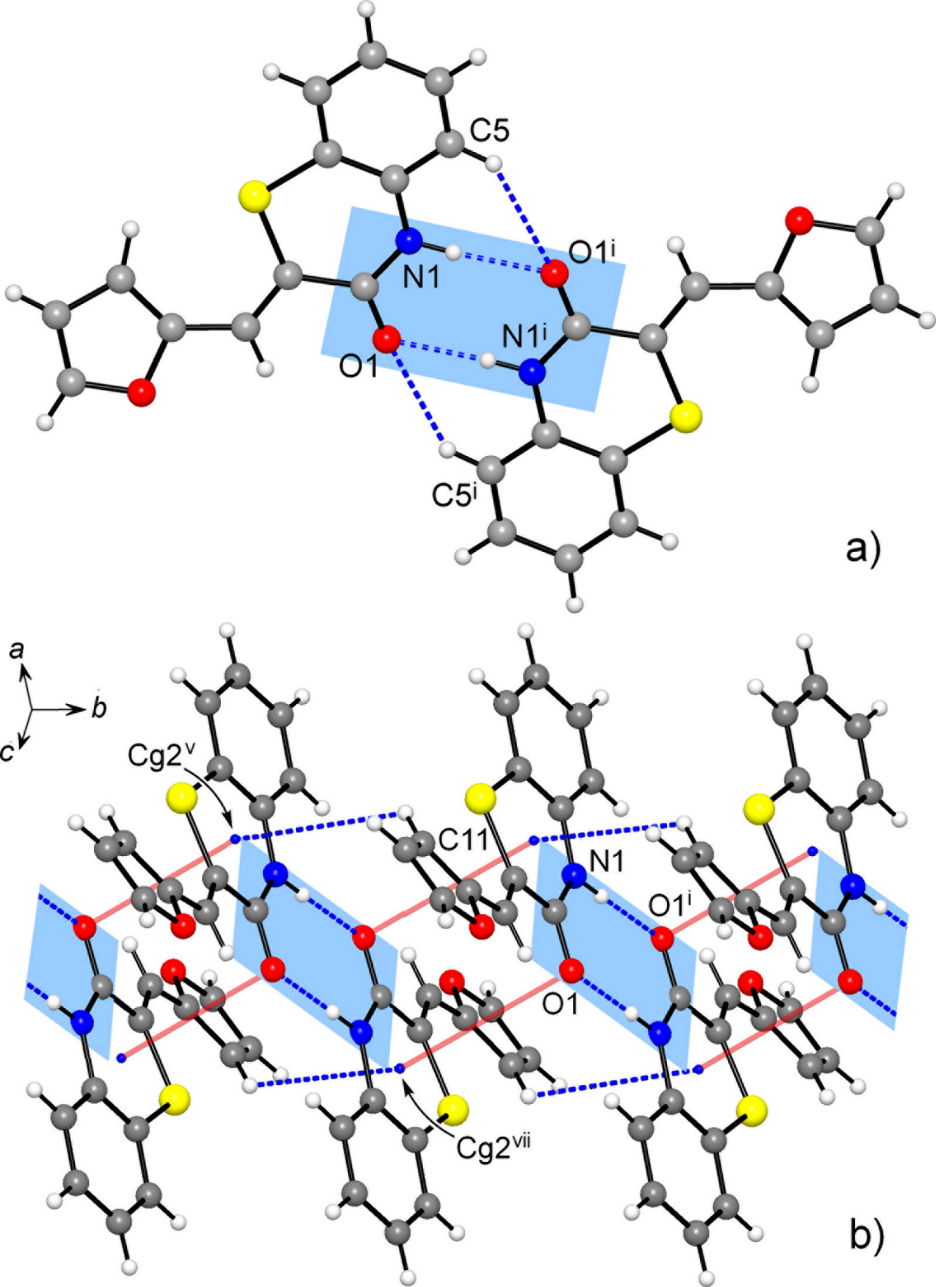
(*a*) Inversion-related hydrogen-bonded dimers in the structure of **2**; (*b*) Columns of stacked dimers, which feature importance of axial inter­actions at the thia­zinone core (red: CO⋯π; blue: C—H⋯π). [Symmetry codes: (i) −*x* + 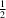, −*y* + 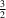, −*z* + 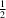; (v) *x*, *y* − 1, *z*; (vii) −*x* + 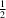, −*y* + 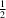, −*x* + 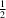.]

**Figure 6:**
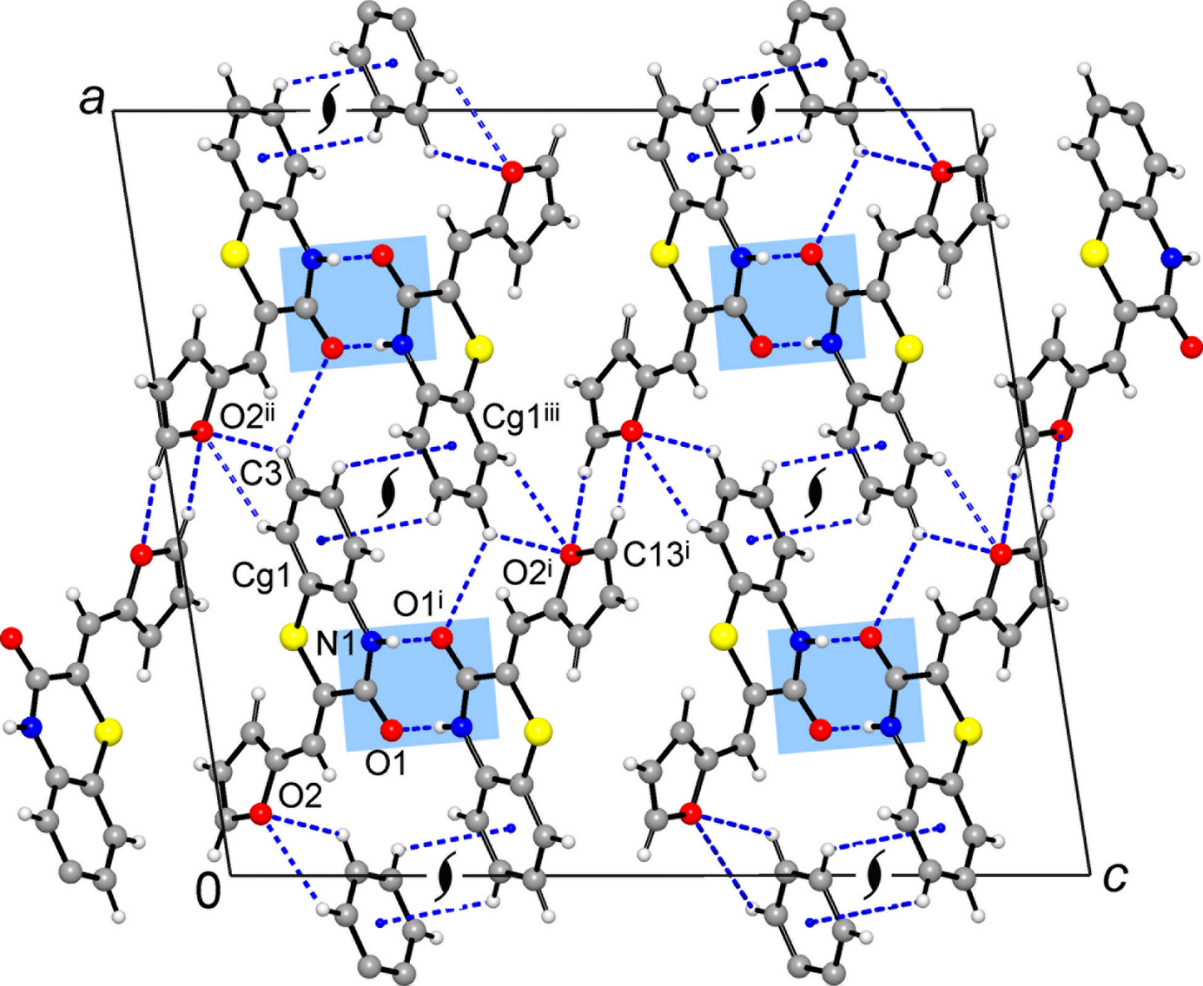
The structure of **2** viewed in a projection on the *ac* plane, showing layers of stacked dimers (orthogonal to the drawing plane) and their connection through mutual C—H⋯O bonding of furyl groups. The orthogonal 2_1_ axes identify helicate configuration of C—H⋯π bonding motif. [Symmetry codes: (i) −*x* + 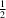, −*y* + 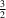, −*z* + 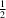; (ii) *x* + 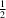, −*y*, *z*; (iii) *x* + 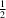, −*y* + 1, *z*.]

The supra­molecular morphology of **2** is very comparable, with some variations conditioned by specific bonding preferences of the furyl ring. Stacking of the dimers yields similar columns, propagating down the *b*-axis direction (Fig. 5 ▸). Instead of the C=O/C=O inter­actions seen in **1**, the mol­ecules afford inversion thia­zinone stacks, with O1⋯*Cg*(S1/N1/C1/C6–C8)^vii^ and O1⋯plane^vii^ distances of 3.2937 (19) and 3.226 (2) Å, respectively [symmetry code: (vii) −*x* + 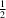, −*y* + 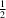, −*z* + 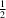]. That the O1 atoms are mutually situated above the adjacent ring centroids is witnessed by the angle between the O1⋯*Cg*^vii^ axis and the ring normal of 11.7 (2)°. This inter­action is accompanied by C—H⋯π bonding between the furyl donors and thia­zinone acceptors (Fig. 5 ▸, Table 2 ▸). Similarly to **1**, the columns are linked into layers (parallel to the *ab* plane) due to C—H⋯π bonds between carbo rings of the benzo­thia­zinone moieties [C4⋯*Cg*(C1–C6) = 3.688 (3) Å], while two additional C—H⋯O bonds occur also with the furyl-O acceptor from a second part of the dimer [C⋯O = 3.420 (3), 3.446 (3) Å; Table 2 ▸]. However, the most notable structural function inherent to the furyl rings is the mutual C—H⋯O bonding, giving inversion dimers with C⋯O = 3.359 (3) A (Fig. 6 ▸). This cyclic pattern itself represents the lowest energy furan dimer calculated for the gas phase (Majerz, 2018 ▸). Such inter­actions provide the connection of the layers into a three-dimensional framework and are particularly essential for structural cohesion as the shortest of the weak hydrogen bonds present with CH donors. One can suppose that a set of supra­molecular inter­actions involving furyl groups may be primarily responsible for the relatively high packing index of 73.4 [*vs*. 71.5 for **1**], which approaches the upper limit of the 65–75% range expected for organic solids (Dunitz, 1995 ▸).

## 4. Hirshfeld analysis

**Figure 7:**
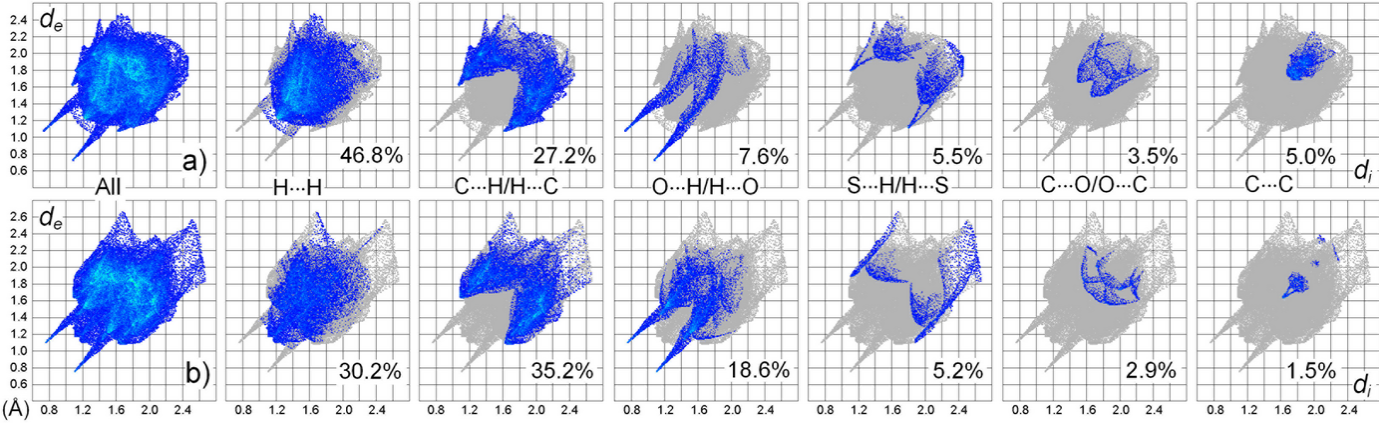
Two-dimensional fingerprint plots for **1** (*a*) and **2** (*b*) for all contacts and delineated into the principal contributions of individual contacts. Other contributions, which account for more than 1.0%, are N⋯H/H⋯N (2.0 and 1.8% for **1** and **2**, respectively) and S⋯C/C⋯S (1.8 and 2.8%).

The supra­molecular inter­actions in the title structures were further assessed by Hirshfeld surface analysis (McKinnon *et al.*, 2007 ▸; Hirshfeld, 1977 ▸; Spackman *et al.*, 2021 ▸) performed with *CrystalExplorer17* (Turner *et al.*, 2017 ▸). The two-dimensional fingerprint plots suggest the dominant role of inter­actions with the H atoms, which account for 71.7 and 65.2% of the contacts in **1** and **2**, respectively. At the same time, there are essential differences due to the replacement of tolyl for furyl groups. Thus, the fractions of C⋯H/H⋯C and O⋯H/H⋯O are expanded from 27.2 and 7.6% in **1** to 35.2 and 18.6% in **2**, primarily at the expense of H⋯H contacts (Fig. 7 ▸). Although this is in line with a larger number of the available O-atom acceptors in the latter case, the ability of the furyl group to maintain multiple weak C—H⋯O inter­actions is also important. The contributions of S⋯H/H⋯S are nearly the same for both compounds and are relatively minor. However, in the case of **1**, one can identify a pair of short spikes pointing to the lower left with *d*_e_ + *d_i_* = 2.95 Å. These features are similar in nature to the short spikes for C⋯H/H⋯C contacts and they likely indicate very weak C—H⋯S hydrogen bonding. For **2**, the S⋯H/H⋯S plot represents rather a collection of points at large *d*_e_ and *d*_i_ distances and moreover, a scarcely populated extended area above *d*_e_ + *d*_i_ = 4.0 Å suggests the existence of small voids around the S atoms. This observation is supported by the volumes of the Dirichlet–Voronoi domains associated with the S1 atoms, which are 48.05 Å^3^ for **1** and 61.41 Å^3^ for **2** and therefore the S1 environment in the latter case is less crowded. Finally, an overlap between nearly parallel mol­ecules, due to the slipped π–π ethene/tolyl stacking, is clearly indicated by the C⋯C plots for **1** (5.0%), in the form of the blue area centred at *ca. d*_e_ = *d*_i_ = 1.80 Å and with a shortest contact of 3.50 Å (Fig. 7 ▸). This feature is only minor in the case of **2**, with a small fraction of slightly shorter C⋯C contacts (1.5%) associated with carbon­yl/thia­zine stacking.

**Figure 8:**
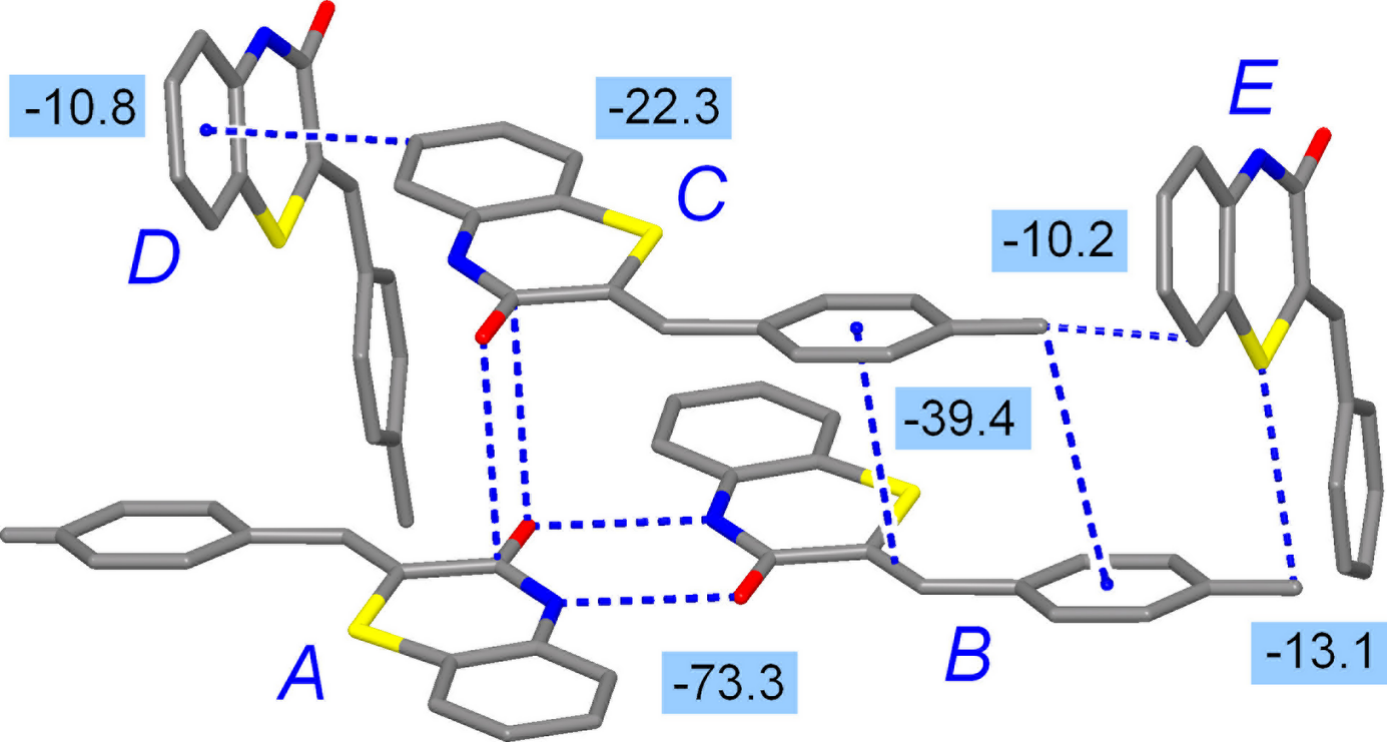
The principal pairwise inter­molecular inter­actions for **1**, identified with a cut-off limit of 10 kJ mol^−1^. The inter­action energies are given in kJ mol^−1^.

The inter­molecular inter­action energies were calculated using the CE B3LYP/6 31G(d,p) energy model in *CrystalExplorer17* (Turner *et al.*, 2017 ▸). With a cut-off of |*E*_tot_| > 10 kJ mol^−1^, five symmetry-independent paths were considered for the closest environment of the mol­ecules in **1** (Table 3 ▸) and the far dominant energy of *E*_tot_ = −73.3 kJ mol^−1^ corresponds to the reciprocal N—H⋯O and C—H⋯O inter­actions within the basic dimer (path *A*⋯*B*, Fig. 8 ▸). This pairing is governed essentially by the electrostatic component (*E*_ele_ = −98.2 kJ mol^−1^) and is very close in energy to inter­actions in 2-pyridone dimers [−68.2 kJ mol^−1^; Inuzuka & Fujimoto, 1982 ▸]. Other structure-defining inter­actions originate in London dispersion, while the most prominent ones are also restricted to the columns of stacked dimers. First, the *B*⋯*C* pair combines tolyl C—H⋯π bonds and π–π stacking within a very large inter­action area. The appreciable resulting energy of −39.4 kJ mol^−1^ is a reflection of a significant dispersion contributor [*E*_dis_ = −62.0 kJ mol^−1^]. Second, mutual anti­parallel stacks of the carbonyl groups (path *A*⋯*C*) are also very favorable with *E*_tot_ = −22.3 kJ mol^−1^, which exactly coincides with the value for model 2-propanone dimers (Allen *et al.*, 1998 ▸).

**Figure 9:**
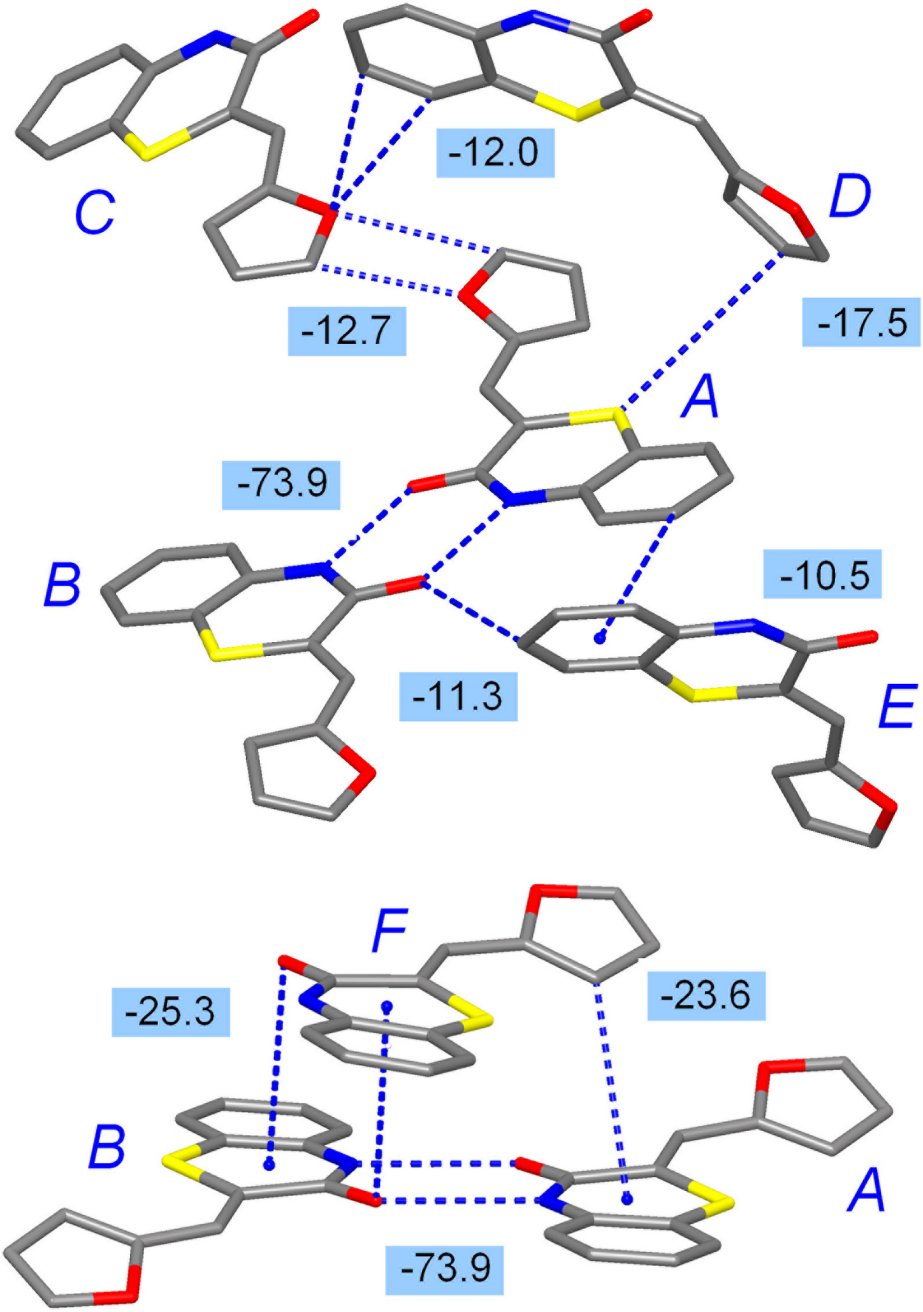
The principal pathways of inter­molecular inter­actions for **2**, identified with a cut-off limit of 10 kJ mol^−1^, which involve different kinds of stacking and hydrogen bonding. The inter­action energies are given in kJ mol^−1^.

The landscape of inter­action energies for **2** is apparently more rich, with nine unique paths above |*E*_tot_| > 10 kJ mol^−1^ (Fig. 9 ▸). In fact, beyond the primary inter­action in the form of electrostatically dominated strong hydrogen bonding (pair *A*⋯*B*, *E*_tot_ = −73.9 kJ mol^−1^), most inter­molecular paths converge in the inter­action energies falling into the −10 to −25 kJ mol^−1^ range (Table 3 ▸). The most prominent inter­actions within this group are mutual carbon­yl–π stacking of path *A*⋯*F* and dispersion and C—H⋯π driven path *B*⋯*F* [*E*_tot_ = −25.3 and −23.6 kJ mol^−1^, respectively]. Both of them are also found within the column of stacked dimers. At the same time, the growing importance of bonding between the subconnectivities of lower dimensionality is best illustrated by the energetics of the furyl dimers established between the layers. The inter­action within the path *A*⋯*C* has comparable electrostatic and dispersion contributors and it results in *E*_tot_ = −12.7 kJ mol^−1^, which is superior to most pairwise inter­actions between the layers in the structure of **1**. The latter value reproduces an energy of −13.0 kJ mol^−1^, found for the doubly C—H⋯O-bonded furan dimer in the gas phase (Majerz, 2018 ▸). One can suppose that the specific behavior of furyl groups in **2**, either as a donor or acceptor of weak C—H⋯O bonding (as may be compared with tolyl groups in **1**) contributes not only to the larger fraction of O⋯H/H⋯O contacts, but also enhances the inter­action energies. Even in spite the possible presence of small crystal voids around the S1 atoms in **2**, the furyl derivative develops a perceptibly higher packing index.

## 5. Database survey

A search of the Cambridge Structural Database (CSD, updated to July 2025; Groom *et al.*, 2016 ▸) for 1,4-benzo­thia­zin-3-one derivatives bearing a substituted methyl­idene fragment at the C^2^-atom and with no substitution at benzo-ring C atoms revealed 18 hits. The group of closest 4-*H* analogs is represented by (*Z*)-2-(1-bromo­ethyl­idine) (CSD refcode BOLDOV; Bates *et al.*, 1982 ▸) and (*Z*)-2-(nitro­methyl­ene) compounds (GETNOJ; Berestovskaya *et al.*, 2006 ▸) and two polymorphs of structurally related thia­zine-indigo (SAJMOH and SAJMOH01; Buchsbaum *et al.*, 2011 ▸), whereas the larger family of 4-*R* derivatives features the incorporation of 2-benzyl­idene and derived fragments, including one example of a 4-methyl­benzyl­idene compound related to the structure of **1** (RURBEO; Sebbar *et al.*, 2020*a* ▸). All these compounds follow the trend established above: the 1,4-thia­zin-3-one core is essentially flat in the case of 4-*H* species, but even 4-methyl substitution (VUXWES; Ellouz *et al.*, 2015 ▸) causes loss of planarity. An appreciable bend of the heteroring may be assessed with values of dihedral angles between the S^1^C^9^C^10^ and N^4^C^3^OC^2^ planes, which are nearly uniform for all 4-*R* compounds within the range 19.2–24.3°. They are systematically much larger than the parameters for 4-*H* species: 5.70 (BOLDOV); 5.82 (GETNOJ); 1.71 (SAJMOH) and 1.51° (SAJMOH01). From a supra­molecular perspective, the comparable examples are restricted to the category of N -species and every such 1,4-benzo­thia­zin-3-one sustains dimeric motifs of reciprocal N—H⋯O inter­actions, which are similar to those in the title structures.

## 6. Synthesis and crystallization

To 300 mg (2.84 mmol) of 3,4-di­hydro-2*H*-1,4-benzo­thia­zin-3-one and 5.68 mmol of either 4-methyl­benzaldehyde (for the synthesis of **1**) or furan-2-carbaldehyde (for the synthesis of **2**) dissolved in 10 ml of anhydrous DMF, 383.4 mg (7.1 mmol) of sodium methoxide were added. The mixture was refluxed for 18 h while being stirred vigorously with a magnetic stirrer. After cooling, the precipitate was filtered out and the filtrate was concentrated under reduced pressure. The resulting crude residue was purified by column chromatography on silica gel using ethyl acetate/hexane (10:90, *v*/*v*) as eluent. Slow evaporation of the collected fractions afforded the pure products: (*Z*)-2-(4-methyl­benzyl­idene)-2*H*-benzo[*b*][1,4]thia­zin-3(4*H*)-one (**1**), obtained as colorless plate-like crystals in 85% yield or 2-(furfuryl­idene)-3,4-di­hydro-2*H*-1,4-benzo­thia­zin-3-one (**2**), obtained as colorless column-like crystals in 79% yield. ^1^H NMR (300 MHz, DMSO-*d*_6_), δ, ppm for **1**: 2.40 (*s*, 3H, CH_3_), 7.02–7.63 (*m*, 8H, Ar–H), 7.80 (*s*, 1H, ethene CH), 11.00 (*s*, 1H, NH). ^1^H NMR (300 MHz, DMSO-*d*_6_), δ, ppm For **2**: 7.63–6.77 (*m*, 7H, Ar–H), 7.98 (*s*, 1H, ethene CH), 11.03 (*s*, 1H, NH).

## 7. Refinement

Crystal data, data collection and structure refinement details are summarized in Table 4 ▸. All hydrogen atoms were located and then freely refined with isotropic displacement parameters, which results in N—H = 0.87 (3) and 0.89 (3); C—H = 0.90 (3)–0.99 (3) and C—H (CH_3_) = 0.94 (4)–0.98 (3) Å.

## Supplementary Material

Crystal structure: contains datablock(s) global, 1, 2. DOI: 10.1107/S2056989025008904/yy2019sup1.cif

Structure factors: contains datablock(s) 1. DOI: 10.1107/S2056989025008904/yy20191sup2.hkl

Supporting information file. DOI: 10.1107/S2056989025008904/yy20191sup4.cml

Structure factors: contains datablock(s) 2. DOI: 10.1107/S2056989025008904/yy20192sup3.hkl

Supporting information file. DOI: 10.1107/S2056989025008904/yy20192sup5.cml

CCDC references: 2494946, 2494945

Additional supporting information:  crystallographic information; 3D view; checkCIF report

## Figures and Tables

**Figure 2:**
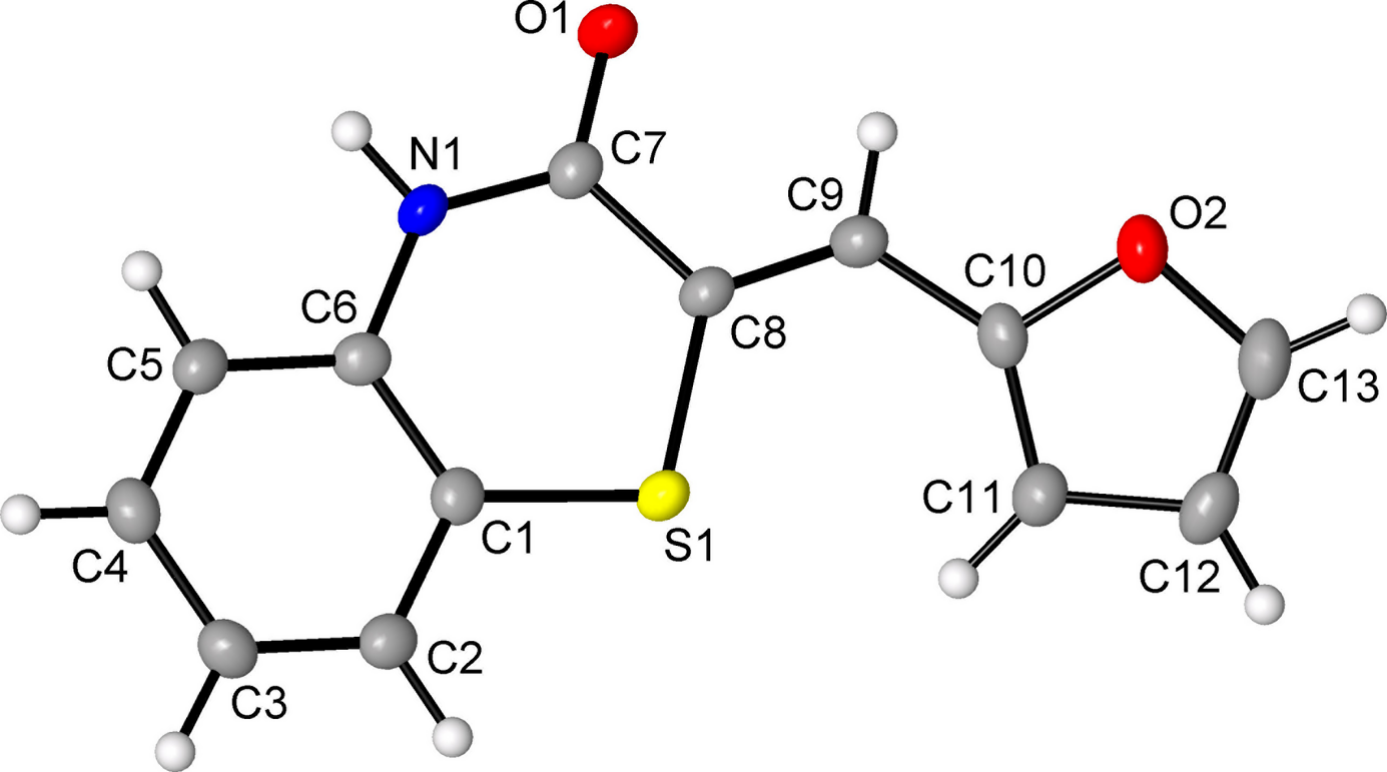
The mol­ecular structure of compound **2**, with atom labelling and displacement ellipsoids drawn at the 50% probability level.

**Table 1:** Hydrogen-bond geometry (Å, °) for **1**[Chem scheme1]

*D*—H⋯*A*	*D*—H	H⋯*A*	*D*⋯*A*	*D*—H⋯*A*
N1—H1*N*⋯O1^i^	0.87 (3)	1.95 (3)	2.822 (2)	177 (3)
C4—H4⋯*Cg*(C1–C6)^ii^	0.98 (2)	2.94 (2)	3.687 (3)	134 (2)
C5—H5⋯O1^i^	0.93 (2)	2.74 (2)	3.440 (3)	132.4 (16)
C16—H16*B*⋯*Cg*(C10–C15)^iii^	0.96 (4)	2.90 (4)	3.813 (3)	158 (2)
C16—H16*C*⋯S1^iv^	0.98 (3)	3.02 (3)	3.865 (3)	146 (2)

Symmetry codes: (i) 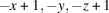; (ii) 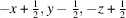; (iii) 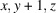; (iv) 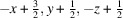.

**Table 2:** Hydrogen-bond geometry (Å, °) for **2**[Chem scheme1]

*D*—H⋯*A*	*D*—H	H⋯*A*	*D*⋯*A*	*D*—H⋯*A*
N1—H1*N*⋯O1^i^	0.89 (3)	2.00 (3)	2.881 (3)	177 (3)
C2—H2⋯O2^ii^	0.96 (3)	2.81 (2)	3.446 (3)	124.2 (19)
C3—H3⋯O1^iii^	0.99 (3)	2.85 (3)	3.573 (3)	130.2 (19)
C3—H3⋯O2^ii^	0.99 (3)	2.78 (3)	3.420 (3)	122.6 (19)
C4—H4⋯*Cg*C1–C6)^iv^	0.97 (3)	3.07 (3)	3.688 (3)	123 (2)
C5—H5⋯O1^i^	0.93 (3)	2.73 (2)	3.490 (3)	138.8 (18)
C11—H11⋯*Cg*(S1/N1/C1/C6–C8)^v^	0.98 (3)	3.06 (3)	3.749 (3)	129 (2)
C13—H13⋯O2^vi^	0.93 (3)	2.68 (3)	3.359 (3)	130 (2)

Symmetry codes: (i) 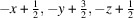; (ii) 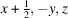; (iii) 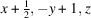; (iv) 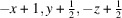; (v) 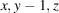; (vi) 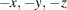.

**Table 3:** Calculated inter­action energies (kJ mol^−1^) Inter­action energies were calculated employing the CE-B3LYP/6–31G(d,p) functional/basis set combination. The scale factors used to determine *E*_tot_: *k*_ele_ = 1.057, *k*_pol_ = 0.740, *k*_dis_ = 0.871, and *k*_rep_ = 0.618 (Mackenzie *et al.*, 2017 ▸). *R* is the distance between the centroids of the inter­acting mol­ecules.

Path	Symmetry code	Type^*a*^	*R* (Å)	*E*_ele_	*E*_pol_	*E*_dis_	*E*_rep_	*E*_tot_
Compound **1**								
*A*⋯*B*	−*x* + 1, −*y*, −*z* + 1	double N—H⋯O and C—H⋯O	8.68	−98.2	−24.6	−21.1	108.7	−73.3
*A*⋯*C*	−*x* + 1, −*y* + 1, −*z* + 1	carbonyl stacking	6.33	−3.8	−3.5	−25.1	10.0	−22.3
*B*′⋯*C*	*x*, *y* − 1, *z*	π–π, C—H⋯π, dispersion	5.19	−8.6	−2.4	−62.0	41.3	−39.4
*B*⋯*E*	−*x* + 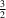 , *y* + 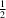 , −*z* + 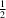	C—H⋯S, dispersion	6.86	−3.5	−0.6	−21.3	15.5	−13.1
*C*⋯*D*	−*x* + 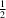 , *y* − 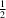 , −*z* + 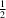	C—H⋯π, dispersion	10.31	−4.0	−0.8	−17.2	14.6	−10.8
*C*⋯*E*	−*x* + 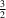 , *y* + 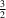 , −*z* + 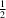	dispersion	10.05	−2.9	−0.4	−13.3	7.7	−10.2
Compound **2**								
*A*⋯*B*	−*x* + 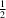 , −*y* + 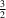 , −*z* + 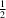	double N—H⋯O and C—H⋯O	7.90	−89.9	−22.3	−19.8	88.9	−73.9
*A*⋯*C*	−*x*, −*y*, −*z*	double C—H⋯O	11.27	−8.4	−1.1	−9.2	7.9	−12.7
*A*⋯*D*	−*x* + 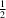 , *y*, −*z*	dispersion	5.26	−2.1	−1.3	−31.2	20.8	−17.5
*A*⋯*E*	−*x* + 1, *y* + 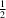 , −*z* + 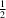	C—H⋯π, dispersion	9.60	−3.1	−0.9	−18.0	14.8	−10.5
*A*⋯*F*	−*x* + 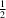 , −*y* + 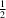 , −*z* + 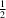	C—H⋯π, dispersion	5.43	−6.6	−1.9	−41.0	30.4	−25.3
*B*⋯*E*	*x* − 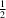 , −*y* + 1, *z*	C—H⋯O	9.93	−4.5	−1.4	−11.9	7.8	−11.3
*B*⋯*F*	*x*, *y* + 1, *z*	stacking	5.44	−3.1	−3.7	−28.9	12.3	−23.6
*C*⋯*D*	*x* − 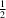 , −*y*, *z*	C—H⋯O, dispersion	9.76	−4.3	−0.7	−13.5	7.9	−12.0

Note: (*a*) For details of the inter­action modes, see Figs. 8 ▸ and 9 ▸.

**Table 4:** Experimental details

	**1**	**2**
Crystal data		
Chemical formula	C_16_H_13_NOS	C_13_H_9_NO_2_S
*M*_r_	267.33	243.27
Crystal system, space group	Monoclinic, *P*2_1_/*n*	Monoclinic, *I*2/*a*
Temperature (K)	150	150
*a*, *b*, *c* (Å)	14.418 (3), 5.1901 (9), 17.425 (3)	18.9170 (5), 5.4280 (2), 20.9874 (7)
β (°)	103.541 (2)	98.702 (1)
*V* (Å^3^)	1267.7 (4)	2130.21 (12)
*Z*	4	8
Radiation type	Mo *K*α	Cu *K*α
μ (mm^−1^)	0.25	2.60
Crystal size (mm)	0.31 × 0.21 × 0.03	0.13 × 0.05 × 0.02

Data collection		
Diffractometer	Bruker SMART APEX CCD	Bruker D8 VENTURE PHOTON 100 CMOS
Absorption correction	Multi-scan (*SADABS*; Krause *et al.*, 2015 ▸)	Multi-scan (*SADABS*; Krause *et al.*, 2015 ▸)
*T*_min_, *T*_max_	0.83, 0.99	0.84, 0.95
No. of measured, independent and observed [*I* > 2σ(*I*)] reflections	11287, 3106, 2192	11794, 1826, 1506
*R*_int_	0.048	0.069
(sin θ/λ)_max_ (Å^−1^)	0.667	0.589

Refinement		
*R*[*F*^2^ > 2σ(*F*^2^)], *wR*(*F*^2^), *S*	0.051, 0.131, 1.04	0.038, 0.090, 1.10
No. of reflections	3106	1826
No. of parameters	224	190
H-atom treatment	All H-atom parameters refined	All H-atom parameters refined
Δρ_max_, Δρ_min_ (e Å^−3^)	0.53, −0.37	0.21, −0.29

Computer programs: *APEX3* and *SAINT* (Bruker, 2016 ▸), *SHELXS97* (Sheldrick, 2008 ▸), *SHELXL2019/3* (Sheldrick, 2015 ▸), *DIAMOND* (Brandenburg, 1999 ▸) and *WinGX* (Farrugia, 2012 ▸).

## supplementary crystallographic information

### (Z)-2-(4-Methylbenzylidene)-2H-benzo[b][1,4]thiazin-3(4H)-one (1) Crystal data

**Table:**

C_16_H_13_NOS	*F*(000) = 560
*M**_r_* = 267.33	*D*_x_ = 1.401 Mg m^−^^3^
Monoclinic, *P*2_1_/*n*	Mo *K*α radiation, λ = 0.71073 Å
*a* = 14.418 (3) Å	Cell parameters from 2851 reflections
*b* = 5.1901 (9) Å	θ = 2.4–27.0°
*c* = 17.425 (3) Å	µ = 0.25 mm^−^^1^
β = 103.541 (2)°	*T* = 150 K
*V* = 1267.7 (4) Å^3^	Plate, colourless
*Z* = 4	0.31 × 0.21 × 0.03 mm

### (Z)-2-(4-Methylbenzylidene)-2H-benzo[b][1,4]thiazin-3(4H)-one (1) Data collection

**Table:**

Bruker SMART APEX CCD diffractometer	3106 independent reflections
Radiation source: fine-focus sealed tube	2192 reflections with *I* > 2σ(*I*)
Graphite monochromator	*R*_int_ = 0.048
Detector resolution: 8.3333 pixels mm^-1^	θ_max_ = 28.3°, θ_min_ = 1.7°
φ and ω scans	*h* = −19→19
Absorption correction: multi-scan (*SADABS*; Krause *et al.*, 2015)	*k* = −6→6
*T*_min_ = 0.83, *T*_max_ = 0.99	*l* = −23→22
11287 measured reflections	

### (Z)-2-(4-Methylbenzylidene)-2H-benzo[b][1,4]thiazin-3(4H)-one (1) Refinement

**Table:**

Refinement on *F*^2^	Primary atom site location: structure-invariant direct methods
Least-squares matrix: full	Secondary atom site location: difference Fourier map
*R*[*F*^2^ > 2σ(*F*^2^)] = 0.051	Hydrogen site location: difference Fourier map
*wR*(*F*^2^) = 0.131	All H-atom parameters refined
*S* = 1.04	*w* = 1/[σ^2^(*F*_o_^2^) + (0.0561*P*)^2^ + 0.425*P*] where *P* = (*F*_o_^2^ + 2*F*_c_^2^)/3
3106 reflections	(Δ/σ)_max_ < 0.001
224 parameters	Δρ_max_ = 0.53 e Å^−^^3^
0 restraints	Δρ_min_ = −0.37 e Å^−^^3^

### (Z)-2-(4-Methylbenzylidene)-2H-benzo[b][1,4]thiazin-3(4H)-one (1) Special details

**Table:**

Experimental. The diffraction data were collected in three sets of 363 frames (0.5° width in ω) at φ = 0, 120 and 240°. A scan time of 80 sec/frame was used.
Geometry. All esds (except the esd in the dihedral angle between two l.s. planes) are estimated using the full covariance matrix. The cell esds are taken into account individually in the estimation of esds in distances, angles and torsion angles; correlations between esds in cell parameters are only used when they are defined by crystal symmetry. An approximate (isotropic) treatment of cell esds is used for estimating esds involving l.s. planes.
Refinement. Refinement of F^2^ against ALL reflections. The weighted R-factor wR and goodness of fit S are based on F^2^, conventional R-factors R are based on F, with F set to zero for negative F^2^. The threshold expression of F^2^ > 2sigma(F^2^) is used only for calculating R-factors(gt) etc. and is not relevant to the choice of reflections for refinement. R-factors based on F^2^ are statistically about twice as large as those based on F, and R- factors based on ALL data will be even larger.

### (Z)-2-(4-Methylbenzylidene)-2H-benzo[b][1,4]thiazin-3(4H)-one (1) Fractional atomic coordinates and isotropic or equivalent isotropic displacement parameters (Å^2^)

**Table:**

	*x*	*y*	*z*	*U*_iso_*/*U*_eq_	
S1	0.55852 (4)	0.55290 (11)	0.30333 (3)	0.03071 (18)	
O1	0.58296 (11)	0.2623 (3)	0.51760 (8)	0.0307 (4)	
N1	0.48480 (13)	0.1456 (4)	0.40334 (10)	0.0268 (4)	
C1	0.46507 (14)	0.3340 (4)	0.27280 (12)	0.0236 (4)	
C2	0.41636 (16)	0.3409 (4)	0.19356 (12)	0.0276 (5)	
C3	0.34365 (16)	0.1682 (4)	0.16488 (13)	0.0310 (5)	
C4	0.31880 (16)	−0.0129 (4)	0.21486 (14)	0.0308 (5)	
C5	0.36606 (15)	−0.0184 (4)	0.29363 (13)	0.0284 (5)	
C6	0.43919 (14)	0.1572 (4)	0.32306 (11)	0.0233 (4)	
C7	0.55369 (15)	0.2991 (4)	0.44557 (12)	0.0241 (4)	
C8	0.59591 (15)	0.5073 (4)	0.40564 (12)	0.0242 (4)	
C9	0.66788 (15)	0.6442 (4)	0.45071 (12)	0.0253 (4)	
C10	0.72877 (14)	0.8460 (4)	0.43109 (12)	0.0246 (4)	
C11	0.71411 (16)	0.9745 (4)	0.35848 (13)	0.0277 (5)	
C12	0.77792 (16)	1.1582 (4)	0.34453 (13)	0.0298 (5)	
C13	0.85867 (16)	1.2238 (4)	0.40155 (12)	0.0290 (5)	
C14	0.87274 (17)	1.1000 (5)	0.47383 (13)	0.0336 (5)	
C15	0.80970 (16)	0.9165 (5)	0.48863 (13)	0.0314 (5)	
C16	0.9272 (2)	1.4253 (5)	0.38564 (15)	0.0366 (6)	
H1N	0.466 (2)	0.019 (5)	0.4291 (17)	0.056 (9)*	
H2	0.4369 (16)	0.468 (4)	0.1593 (14)	0.030 (6)*	
H3	0.3106 (17)	0.170 (4)	0.1096 (14)	0.036 (6)*	
H4	0.2677 (15)	−0.136 (4)	0.1952 (12)	0.023 (5)*	
H5	0.3464 (15)	−0.137 (4)	0.3269 (13)	0.026 (6)*	
H9	0.6836 (16)	0.600 (4)	0.5040 (14)	0.028 (6)*	
H11	0.6590 (17)	0.940 (4)	0.3189 (14)	0.034 (6)*	
H12	0.7653 (16)	1.240 (4)	0.2961 (14)	0.032 (6)*	
H14	0.9262 (19)	1.134 (5)	0.5113 (15)	0.043 (7)*	
H15	0.8185 (17)	0.837 (5)	0.5372 (15)	0.037 (7)*	
H16A	0.989 (3)	1.390 (7)	0.417 (2)	0.097 (13)*	
H16B	0.909 (3)	1.603 (8)	0.389 (2)	0.102 (13)*	
H16C	0.937 (2)	1.410 (6)	0.3321 (19)	0.066 (9)*	

### (Z)-2-(4-Methylbenzylidene)-2H-benzo[b][1,4]thiazin-3(4H)-one (1) Atomic displacement parameters (Å^2^)

**Table:**

	*U*^11^	*U*^22^	*U*^33^	*U*^12^	*U*^13^	*U*^23^
S1	0.0319 (3)	0.0350 (3)	0.0211 (3)	−0.0111 (2)	−0.0021 (2)	0.0073 (2)
O1	0.0343 (9)	0.0342 (8)	0.0217 (7)	−0.0073 (7)	0.0025 (6)	0.0067 (6)
N1	0.0283 (9)	0.0288 (10)	0.0216 (9)	−0.0042 (8)	0.0022 (7)	0.0072 (7)
C1	0.0225 (10)	0.0229 (10)	0.0240 (10)	−0.0004 (8)	0.0027 (8)	0.0010 (8)
C2	0.0301 (11)	0.0287 (11)	0.0224 (10)	0.0000 (9)	0.0031 (9)	0.0021 (8)
C3	0.0316 (12)	0.0340 (12)	0.0241 (11)	−0.0007 (10)	0.0002 (9)	−0.0029 (9)
C4	0.0274 (12)	0.0280 (12)	0.0343 (12)	−0.0031 (9)	0.0019 (10)	−0.0025 (9)
C5	0.0261 (11)	0.0272 (11)	0.0312 (11)	−0.0017 (9)	0.0052 (9)	0.0034 (9)
C6	0.0217 (10)	0.0253 (10)	0.0220 (10)	0.0025 (8)	0.0034 (8)	0.0024 (8)
C7	0.0244 (10)	0.0241 (10)	0.0235 (10)	−0.0004 (8)	0.0048 (8)	0.0037 (8)
C8	0.0247 (10)	0.0255 (11)	0.0219 (10)	0.0019 (8)	0.0046 (8)	0.0042 (8)
C9	0.0273 (11)	0.0271 (11)	0.0206 (10)	0.0001 (9)	0.0041 (8)	0.0030 (8)
C10	0.0249 (10)	0.0260 (11)	0.0232 (10)	−0.0022 (9)	0.0063 (8)	−0.0027 (8)
C11	0.0252 (11)	0.0318 (12)	0.0240 (10)	−0.0045 (9)	0.0019 (9)	−0.0010 (9)
C12	0.0353 (12)	0.0298 (11)	0.0246 (11)	−0.0038 (10)	0.0074 (9)	0.0009 (9)
C13	0.0331 (12)	0.0275 (11)	0.0288 (11)	−0.0053 (9)	0.0121 (10)	−0.0082 (9)
C14	0.0332 (13)	0.0411 (13)	0.0237 (11)	−0.0112 (10)	0.0011 (10)	−0.0072 (9)
C15	0.0362 (13)	0.0374 (13)	0.0188 (10)	−0.0068 (10)	0.0032 (9)	−0.0009 (9)
C16	0.0410 (15)	0.0359 (14)	0.0360 (13)	−0.0163 (12)	0.0150 (12)	−0.0100 (11)

### (Z)-2-(4-Methylbenzylidene)-2H-benzo[b][1,4]thiazin-3(4H)-one (1) Geometric parameters (Å, º)

**Table:**

S1—C1	1.747 (2)	C8—C9	1.348 (3)
S1—C8	1.754 (2)	C9—C10	1.458 (3)
O1—C7	1.242 (2)	C9—H9	0.93 (2)
N1—C7	1.350 (3)	C10—C15	1.397 (3)
N1—C6	1.401 (2)	C10—C11	1.402 (3)
N1—H1N	0.87 (3)	C11—C12	1.385 (3)
C1—C6	1.379 (3)	C11—H11	0.94 (2)
C1—C2	1.395 (3)	C12—C13	1.384 (3)
C2—C3	1.381 (3)	C12—H12	0.92 (2)
C2—H2	0.98 (2)	C13—C14	1.386 (3)
C3—C4	1.384 (3)	C13—C16	1.508 (3)
C3—H3	0.97 (2)	C14—C15	1.382 (3)
C4—C5	1.382 (3)	C14—H14	0.90 (3)
C4—H4	0.98 (2)	C15—H15	0.92 (2)
C5—C6	1.397 (3)	C16—H16A	0.94 (4)
C5—H5	0.93 (2)	C16—H16B	0.96 (4)
C7—C8	1.490 (3)	C16—H16C	0.98 (3)
			
C1—S1—C8	104.68 (10)	C8—C9—C10	131.7 (2)
C7—N1—C6	129.24 (19)	C8—C9—H9	115.3 (14)
C7—N1—H1N	116.1 (19)	C10—C9—H9	112.9 (14)
C6—N1—H1N	114.7 (19)	C15—C10—C11	116.56 (19)
C6—C1—C2	119.82 (19)	C15—C10—C9	117.86 (19)
C6—C1—S1	122.91 (15)	C11—C10—C9	125.57 (19)
C2—C1—S1	117.27 (16)	C12—C11—C10	121.3 (2)
C3—C2—C1	120.3 (2)	C12—C11—H11	119.0 (14)
C3—C2—H2	121.9 (13)	C10—C11—H11	119.7 (14)
C1—C2—H2	117.7 (13)	C13—C12—C11	121.6 (2)
C2—C3—C4	120.0 (2)	C13—C12—H12	119.7 (15)
C2—C3—H3	120.5 (14)	C11—C12—H12	118.7 (15)
C4—C3—H3	119.5 (14)	C12—C13—C14	117.3 (2)
C5—C4—C3	119.9 (2)	C12—C13—C16	121.0 (2)
C5—C4—H4	119.4 (12)	C14—C13—C16	121.7 (2)
C3—C4—H4	120.7 (12)	C15—C14—C13	121.8 (2)
C4—C5—C6	120.3 (2)	C15—C14—H14	119.2 (16)
C4—C5—H5	118.7 (13)	C13—C14—H14	118.9 (17)
C6—C5—H5	120.9 (13)	C14—C15—C10	121.4 (2)
C1—C6—C5	119.61 (18)	C14—C15—H15	121.7 (15)
C1—C6—N1	121.97 (19)	C10—C15—H15	116.9 (15)
C5—C6—N1	118.41 (19)	C13—C16—H16A	109 (2)
O1—C7—N1	119.53 (18)	C13—C16—H16B	117 (2)
O1—C7—C8	120.28 (18)	H16A—C16—H16B	112 (3)
N1—C7—C8	120.17 (18)	C13—C16—H16C	111.5 (18)
C9—C8—C7	116.94 (18)	H16A—C16—H16C	102 (3)
C9—C8—S1	122.19 (16)	H16B—C16—H16C	104 (3)
C7—C8—S1	120.71 (15)		
			
C8—S1—C1—C6	−5.2 (2)	O1—C7—C8—S1	177.44 (16)
C8—S1—C1—C2	175.45 (17)	N1—C7—C8—S1	−1.1 (3)
C6—C1—C2—C3	−1.3 (3)	C1—S1—C8—C9	−179.98 (18)
S1—C1—C2—C3	178.04 (17)	C1—S1—C8—C7	4.81 (19)
C1—C2—C3—C4	0.0 (3)	C7—C8—C9—C10	175.5 (2)
C2—C3—C4—C5	0.8 (3)	S1—C8—C9—C10	0.1 (4)
C3—C4—C5—C6	−0.3 (3)	C8—C9—C10—C15	−167.7 (2)
C2—C1—C6—C5	1.8 (3)	C8—C9—C10—C11	11.2 (4)
S1—C1—C6—C5	−177.52 (16)	C15—C10—C11—C12	1.2 (3)
C2—C1—C6—N1	−178.91 (19)	C9—C10—C11—C12	−177.8 (2)
S1—C1—C6—N1	1.8 (3)	C10—C11—C12—C13	−0.2 (3)
C4—C5—C6—C1	−1.0 (3)	C11—C12—C13—C14	−0.7 (3)
C4—C5—C6—N1	179.7 (2)	C11—C12—C13—C16	−179.6 (2)
C7—N1—C6—C1	3.7 (3)	C12—C13—C14—C15	0.7 (4)
C7—N1—C6—C5	−177.0 (2)	C16—C13—C14—C15	179.6 (2)
C6—N1—C7—O1	177.5 (2)	C13—C14—C15—C10	0.3 (4)
C6—N1—C7—C8	−4.0 (3)	C11—C10—C15—C14	−1.2 (3)
O1—C7—C8—C9	2.0 (3)	C9—C10—C15—C14	177.8 (2)
N1—C7—C8—C9	−176.53 (19)		

### (Z)-2-(4-Methylbenzylidene)-2H-benzo[b][1,4]thiazin-3(4H)-one (1) Hydrogen-bond geometry (Å, º)

**Table:**

*D*—H···*A*	*D*—H	H···*A*	*D*···*A*	*D*—H···*A*
N1—H1*N*···O1^i^	0.87 (3)	1.95 (3)	2.822 (2)	177 (3)
C4—H4···*Cg*(C1–C6)^ii^	0.98 (2)	2.94 (2)	3.687 (3)	134 (2)
C5—H5···O1^i^	0.93 (2)	2.74 (2)	3.440 (3)	132.4 (16)
C16—H16*B*···*Cg*(C10–C15)^iii^	0.96 (4)	2.90 (4)	3.813 (3)	158 (2)
C16—H16*C*···S1^iv^	0.98 (3)	3.02 (3)	3.865 (3)	146 (2)

Symmetry codes: (i) −*x*+1, −*y*, −*z*+1; (ii) −*x*+1/2, *y*−1/2, −*z*+1/2; (iii) *x*, *y*+1, *z*; (iv) −*x*+3/2, *y*+1/2, −*z*+1/2.

### (Z)-2-(Furan-2-ylmethylidene)-2H-benzo[b][1,4]thiazin-3(4H)-one (2) Crystal data

**Table:**

C_13_H_9_NO_2_S	*F*(000) = 1008
*M**_r_* = 243.27	*D*_x_ = 1.517 Mg m^−^^3^
Monoclinic, *I*2/*a*	Cu *K*α radiation, λ = 1.54178 Å
*a* = 18.9170 (5) Å	Cell parameters from 7352 reflections
*b* = 5.4280 (2) Å	θ = 4.3–65.2°
*c* = 20.9874 (7) Å	µ = 2.60 mm^−^^1^
β = 98.702 (1)°	*T* = 150 K
*V* = 2130.21 (12) Å^3^	Column, colourless
*Z* = 8	0.13 × 0.05 × 0.02 mm

### (Z)-2-(Furan-2-ylmethylidene)-2H-benzo[b][1,4]thiazin-3(4H)-one (2) Data collection

**Table:**

Bruker D8 VENTURE PHOTON 100 CMOS diffractometer	1826 independent reflections
Radiation source: INCOATEC IµS micro–focus source	1506 reflections with *I* > 2σ(*I*)
Mirror monochromator	*R*_int_ = 0.069
Detector resolution: 10.4167 pixels mm^-1^	θ_max_ = 65.2°, θ_min_ = 4.3°
ω scans	*h* = −22→22
Absorption correction: multi-scan (*SADABS*; Krause *et al.*, 2015)	*k* = −6→6
*T*_min_ = 0.84, *T*_max_ = 0.95	*l* = −24→24
11794 measured reflections	

### (Z)-2-(Furan-2-ylmethylidene)-2H-benzo[b][1,4]thiazin-3(4H)-one (2) Refinement

**Table:**

Refinement on *F*^2^	Primary atom site location: structure-invariant direct methods
Least-squares matrix: full	Secondary atom site location: difference Fourier map
*R*[*F*^2^ > 2σ(*F*^2^)] = 0.038	Hydrogen site location: difference Fourier map
*wR*(*F*^2^) = 0.090	All H-atom parameters refined
*S* = 1.10	*w* = 1/[σ^2^(*F*_o_^2^) + (0.0382*P*)^2^ + 2.4209*P*] where *P* = (*F*_o_^2^ + 2*F*_c_^2^)/3
1826 reflections	(Δ/σ)_max_ < 0.001
190 parameters	Δρ_max_ = 0.21 e Å^−^^3^
0 restraints	Δρ_min_ = −0.28 e Å^−^^3^

### (Z)-2-(Furan-2-ylmethylidene)-2H-benzo[b][1,4]thiazin-3(4H)-one (2) Special details

**Table:**

Geometry. All esds (except the esd in the dihedral angle between two l.s. planes) are estimated using the full covariance matrix. The cell esds are taken into account individually in the estimation of esds in distances, angles and torsion angles; correlations between esds in cell parameters are only used when they are defined by crystal symmetry. An approximate (isotropic) treatment of cell esds is used for estimating esds involving l.s. planes.

### (Z)-2-(Furan-2-ylmethylidene)-2H-benzo[b][1,4]thiazin-3(4H)-one (2) Fractional atomic coordinates and isotropic or equivalent isotropic displacement parameters (Å^2^)

**Table:**

	*x*	*y*	*z*	*U*_iso_*/*U*_eq_	
S1	0.31212 (3)	0.10632 (11)	0.11631 (3)	0.02437 (19)	
O1	0.18959 (8)	0.5139 (3)	0.21259 (8)	0.0278 (4)	
O2	0.08078 (8)	−0.0479 (3)	0.04622 (8)	0.0290 (4)	
N1	0.30581 (10)	0.5594 (4)	0.20528 (9)	0.0207 (4)	
C1	0.38122 (12)	0.3098 (4)	0.14519 (10)	0.0192 (5)	
C2	0.44861 (12)	0.2685 (4)	0.12735 (11)	0.0221 (5)	
C3	0.50469 (12)	0.4255 (5)	0.14732 (11)	0.0232 (5)	
C4	0.49456 (13)	0.6284 (5)	0.18555 (11)	0.0249 (5)	
C5	0.42823 (12)	0.6707 (4)	0.20361 (11)	0.0219 (5)	
C6	0.37169 (12)	0.5111 (4)	0.18414 (10)	0.0192 (5)	
C7	0.24287 (12)	0.4442 (4)	0.18943 (10)	0.0203 (5)	
C8	0.23628 (11)	0.2355 (4)	0.14215 (10)	0.0188 (5)	
C9	0.17072 (13)	0.1519 (4)	0.11978 (11)	0.0224 (5)	
C10	0.15189 (12)	−0.0431 (4)	0.07434 (11)	0.0219 (5)	
C11	0.18668 (13)	−0.2345 (4)	0.05112 (11)	0.0243 (5)	
C12	0.13564 (14)	−0.3639 (5)	0.00687 (12)	0.0291 (6)	
C13	0.07351 (14)	−0.2461 (5)	0.00537 (12)	0.0302 (6)	
H1N	0.3056 (15)	0.691 (6)	0.2301 (15)	0.046 (9)*	
H2	0.4560 (13)	0.129 (5)	0.1012 (12)	0.030 (7)*	
H3	0.5534 (15)	0.393 (5)	0.1376 (13)	0.034 (7)*	
H4	0.5335 (14)	0.741 (5)	0.1993 (12)	0.029 (7)*	
H5	0.4188 (12)	0.807 (5)	0.2282 (12)	0.020 (6)*	
H9	0.1318 (13)	0.231 (5)	0.1329 (11)	0.021 (6)*	
H11	0.2378 (15)	−0.266 (5)	0.0648 (13)	0.033 (7)*	
H12	0.1460 (16)	−0.504 (6)	−0.0150 (15)	0.046 (8)*	
H13	0.0271 (15)	−0.272 (5)	−0.0154 (14)	0.040 (8)*	

### (Z)-2-(Furan-2-ylmethylidene)-2H-benzo[b][1,4]thiazin-3(4H)-one (2) Atomic displacement parameters (Å^2^)

**Table:**

	*U*^11^	*U*^22^	*U*^33^	*U*^12^	*U*^13^	*U*^23^
S1	0.0212 (3)	0.0225 (3)	0.0298 (3)	−0.0008 (2)	0.0052 (2)	−0.0102 (2)
O1	0.0239 (9)	0.0300 (10)	0.0315 (9)	−0.0028 (7)	0.0111 (7)	−0.0116 (7)
O2	0.0231 (9)	0.0302 (10)	0.0321 (9)	−0.0055 (7)	−0.0006 (7)	−0.0064 (7)
N1	0.0220 (10)	0.0182 (10)	0.0218 (10)	−0.0014 (8)	0.0033 (8)	−0.0067 (8)
C1	0.0217 (11)	0.0199 (12)	0.0155 (10)	0.0000 (9)	0.0008 (9)	0.0017 (9)
C2	0.0236 (12)	0.0227 (12)	0.0195 (11)	0.0025 (10)	0.0017 (9)	−0.0011 (10)
C3	0.0200 (12)	0.0279 (13)	0.0219 (12)	0.0008 (10)	0.0031 (9)	0.0015 (10)
C4	0.0213 (12)	0.0267 (13)	0.0259 (12)	−0.0034 (11)	0.0009 (10)	0.0003 (10)
C5	0.0235 (12)	0.0210 (12)	0.0206 (11)	0.0009 (10)	0.0015 (10)	−0.0029 (10)
C6	0.0223 (11)	0.0192 (11)	0.0165 (11)	0.0017 (10)	0.0043 (9)	0.0020 (9)
C7	0.0248 (12)	0.0191 (12)	0.0172 (11)	−0.0010 (10)	0.0037 (9)	−0.0007 (9)
C8	0.0226 (11)	0.0172 (12)	0.0173 (11)	0.0000 (9)	0.0050 (9)	−0.0017 (9)
C9	0.0221 (12)	0.0209 (12)	0.0251 (12)	0.0004 (10)	0.0064 (10)	−0.0027 (10)
C10	0.0200 (11)	0.0252 (13)	0.0199 (11)	−0.0057 (10)	0.0014 (9)	0.0008 (10)
C11	0.0260 (12)	0.0235 (13)	0.0231 (12)	−0.0030 (11)	0.0028 (10)	−0.0024 (10)
C12	0.0378 (15)	0.0267 (14)	0.0234 (12)	−0.0078 (12)	0.0061 (11)	−0.0060 (11)
C13	0.0330 (14)	0.0308 (15)	0.0253 (13)	−0.0115 (12)	−0.0006 (11)	−0.0021 (11)

### (Z)-2-(Furan-2-ylmethylidene)-2H-benzo[b][1,4]thiazin-3(4H)-one (2) Geometric parameters (Å, º)

**Table:**

S1—C1	1.748 (2)	C4—C5	1.384 (3)
S1—C8	1.755 (2)	C4—H4	0.97 (3)
O1—C7	1.242 (3)	C5—C6	1.389 (3)
O2—C13	1.370 (3)	C5—H5	0.93 (3)
O2—C10	1.385 (3)	C7—C8	1.499 (3)
N1—C7	1.342 (3)	C8—C9	1.337 (3)
N1—C6	1.409 (3)	C9—C10	1.433 (3)
N1—H1N	0.89 (3)	C9—H9	0.93 (2)
C1—C6	1.393 (3)	C10—C11	1.359 (3)
C1—C2	1.400 (3)	C11—C12	1.420 (3)
C2—C3	1.376 (3)	C11—H11	0.98 (3)
C2—H2	0.96 (3)	C12—C13	1.334 (4)
C3—C4	1.393 (3)	C12—H12	0.93 (3)
C3—H3	0.99 (3)	C13—H13	0.93 (3)
			
C1—S1—C8	104.12 (11)	C1—C6—N1	122.0 (2)
C13—O2—C10	106.29 (19)	O1—C7—N1	120.2 (2)
C7—N1—C6	129.0 (2)	O1—C7—C8	119.9 (2)
C7—N1—H1N	116.2 (19)	N1—C7—C8	119.90 (19)
C6—N1—H1N	114.7 (19)	C9—C8—C7	118.0 (2)
C6—C1—C2	119.1 (2)	C9—C8—S1	120.93 (17)
C6—C1—S1	122.87 (17)	C7—C8—S1	121.11 (16)
C2—C1—S1	118.01 (17)	C8—C9—C10	127.5 (2)
C3—C2—C1	120.7 (2)	C8—C9—H9	118.3 (15)
C3—C2—H2	119.5 (15)	C10—C9—H9	114.1 (15)
C1—C2—H2	119.8 (15)	C11—C10—O2	109.0 (2)
C2—C3—C4	119.9 (2)	C11—C10—C9	135.9 (2)
C2—C3—H3	121.7 (16)	O2—C10—C9	115.2 (2)
C4—C3—H3	118.3 (16)	C10—C11—C12	107.1 (2)
C5—C4—C3	119.9 (2)	C10—C11—H11	122.5 (16)
C5—C4—H4	119.7 (15)	C12—C11—H11	130.4 (16)
C3—C4—H4	120.4 (15)	C13—C12—C11	106.7 (2)
C4—C5—C6	120.4 (2)	C13—C12—H12	129 (2)
C4—C5—H5	122.7 (15)	C11—C12—H12	124 (2)
C6—C5—H5	116.9 (15)	C12—C13—O2	110.9 (2)
C5—C6—C1	120.0 (2)	C12—C13—H13	134.7 (18)
C5—C6—N1	118.0 (2)	O2—C13—H13	114.3 (18)
			
C8—S1—C1—C6	4.7 (2)	O1—C7—C8—C9	8.7 (3)
C8—S1—C1—C2	−174.43 (17)	N1—C7—C8—C9	−169.4 (2)
C6—C1—C2—C3	−0.7 (3)	O1—C7—C8—S1	−171.80 (17)
S1—C1—C2—C3	178.49 (18)	N1—C7—C8—S1	10.0 (3)
C1—C2—C3—C4	−0.2 (3)	C1—S1—C8—C9	168.76 (19)
C2—C3—C4—C5	0.3 (4)	C1—S1—C8—C7	−10.7 (2)
C3—C4—C5—C6	0.4 (4)	C7—C8—C9—C10	179.5 (2)
C4—C5—C6—C1	−1.2 (3)	S1—C8—C9—C10	0.1 (4)
C4—C5—C6—N1	178.4 (2)	C13—O2—C10—C11	−0.2 (2)
C2—C1—C6—C5	1.4 (3)	C13—O2—C10—C9	−179.4 (2)
S1—C1—C6—C5	−177.75 (17)	C8—C9—C10—C11	18.1 (4)
C2—C1—C6—N1	−178.2 (2)	C8—C9—C10—O2	−162.9 (2)
S1—C1—C6—N1	2.6 (3)	O2—C10—C11—C12	0.0 (3)
C7—N1—C6—C5	174.8 (2)	C9—C10—C11—C12	179.0 (3)
C7—N1—C6—C1	−5.6 (4)	C10—C11—C12—C13	0.1 (3)
C6—N1—C7—O1	−179.4 (2)	C11—C12—C13—O2	−0.2 (3)
C6—N1—C7—C8	−1.2 (3)	C10—O2—C13—C12	0.3 (3)

### (Z)-2-(Furan-2-ylmethylidene)-2H-benzo[b][1,4]thiazin-3(4H)-one (2) Hydrogen-bond geometry (Å, º)

**Table:**

*D*—H···*A*	*D*—H	H···*A*	*D*···*A*	*D*—H···*A*
N1—H1*N*···O1^i^	0.89 (3)	2.00 (3)	2.881 (3)	177 (3)
C2—H2···O2^ii^	0.96 (3)	2.81 (2)	3.446 (3)	124.2 (19)
C3—H3···O1^iii^	0.99 (3)	2.85 (3)	3.573 (3)	130.2 (19)
C3—H3···O2^ii^	0.99 (3)	2.78 (3)	3.420 (3)	122.6 (19)
C4—H4···*Cg*C1–C6)^iv^	0.97 (3)	3.07 (3)	3.688 (3)	123 (2)
C5—H5···O1^i^	0.93 (3)	2.73 (2)	3.490 (3)	138.8 (18)
C11—H11···*Cg*(S1/N1/C1/C6–C8)^v^	0.98 (3)	3.06 (3)	3.749 (3)	129 (2)
C13—H13···O2^vi^	0.93 (3)	2.68 (3)	3.359 (3)	130 (2)

Symmetry codes: (i) −*x*+1/2, −*y*+3/2, −*z*+1/2; (ii) *x*+1/2, −*y*, *z*; (iii) *x*+1/2, −*y*+1, *z*; (iv) −*x*+1, *y*+1/2, −*z*+1/2; (v) *x*, *y*−1, *z*; (vi) −*x*, −*y*, −*z*.
